# Supplementary material for: Tailoring the Electrical Energy Storage Capability of Dielectric Polymer Nanocomposites via Engineering of the Host–Guest Interface by Phosphonic Acids
Source: Molecules. 2022 Oct 25;27(21):7225. doi: 10.3390/molecules27217225 (PMC9654262; doi:10.3390/molecules27217225)
Supplement: Supplementary file 1 [file molecules-27-07225-s001.zip › molecules-1967789-supplementary.pdf]

SUPPLEMENTARY INFORMATION

Tailoring the Electrical Energy Storage Capability of Dielectric Polymers Nanocomposites via Engineering the Host-Guest Interface by Phosphonic Acids

Shaojing Wang[1], Peng Xu[1], Xiangyi Xu[1], Da Kang[2], Jie Chen[2]*, Zhe Li[3]*, Xingyi Huang[2]

[1]State Grid Shanghai Municipal Electric Power Company, Shanghai 200437, China

[2]Shanghai Key Lab of Electrical Insulation and Thermal Aging, Shanghai Jiao Tong University, Shanghai 200240, China

[3]Department of Electrical Engineering, Shanghai Jiao Tong University, Shanghai 200240, China

1. SURFACE MODIFICATION OF BT NANOPARTICLES BY PHOSPHONIC ACIDS

Phosphonic acids with different functional groups were used to treat BT nanoparticles, a typical procedure was as follows: 0.4 g BT particles were dispersed by sonication in 10 mL mixture solvent of ethanol/water. 0.2 mmol BPA, FPMPA, PFBPA, NOPA, HPA and ODPA were dissolved in 1 mL mixture solvent of ethanol/water with a volume ratio of 95:5, respectively. Then, phosphonic acid solutions were added into BT/ethanol/water suspension. After that, the mixture was placed in a sonication bath for 20 min and stirred for 12 h at 80 °C. The nanoparticles were collected by centrifugation at 9000 rpm for 20 min and washed with ethanol for 3 or 4 times. The modified BT nanoparticles were obtained after drying at vacuum overnight. These six modified BT were named as BPA@BT, FPMPA@BT, PFBPA@BT, NOPA@BT, HPA@BT, ODPA@BT, respectively. The mechanism of phosphonic acids binging to BT is shown in Figure 1.

2. PREPARATION OF COMPOSITES

The typical preparation of phosphonic acids modified BT composite films were carried out as follows: A certain amount of modified BT nanoparticles were dispersed in 5 mL DMF by ultrasonication and vigorous stirring for 24 h at room temperature. 1 g P(VDF-HFP) was added into modified BT suspension, ultrasonication for 1 h and vigorous stirring for 24 h at room temperature. After that, the mixture was casted onto a glass plate through a doctor blade and dried in vacuum oven at 40 °C for 24 h. The glass plate with nanocomposite film was put into a 200 °C oven for 5 min and quenched in ice water to increase the nonpolar γ-phase in the polymer matrix. The obtained film was dried at 40 °C for 12 h, and the final film was about 15 μm thick. P(VDF-HFP) nanocomposite films with different volume fractions (5, 10, 15 and 20 vol %) of modified BT were prepared by solution blending.

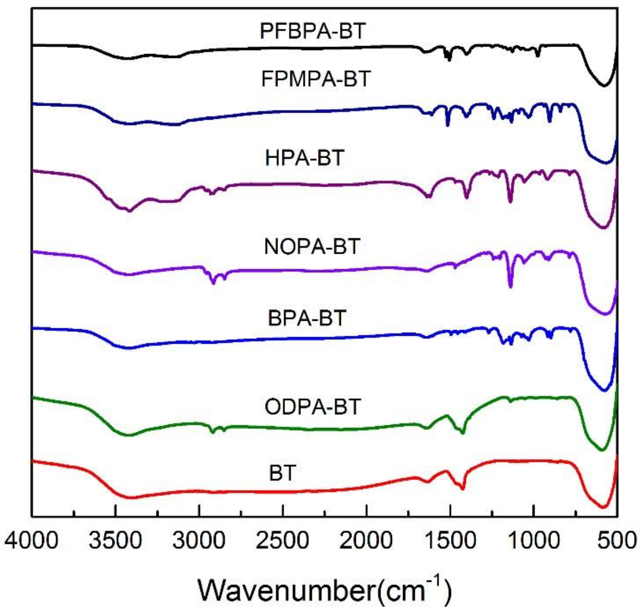

Figure S1. The FT-IR spectra of HPA-BT、NOPA-BT、ODPA-BT、BPA-BT、FPMPA-BT, PFBPA-BT and unmodified BT

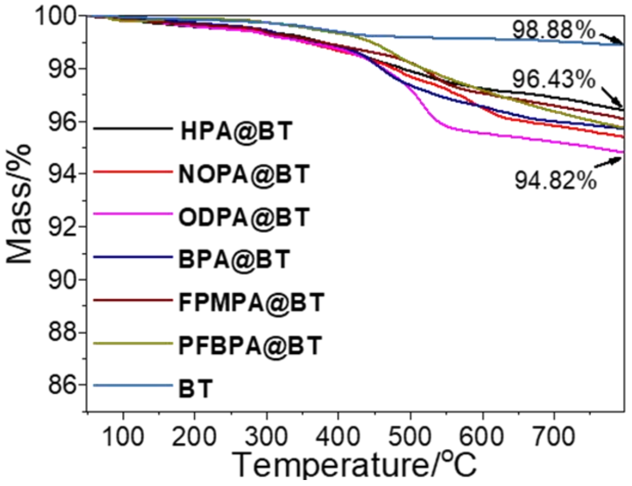

Figure S2. TGA curves of phosphoric acids modified BTO.

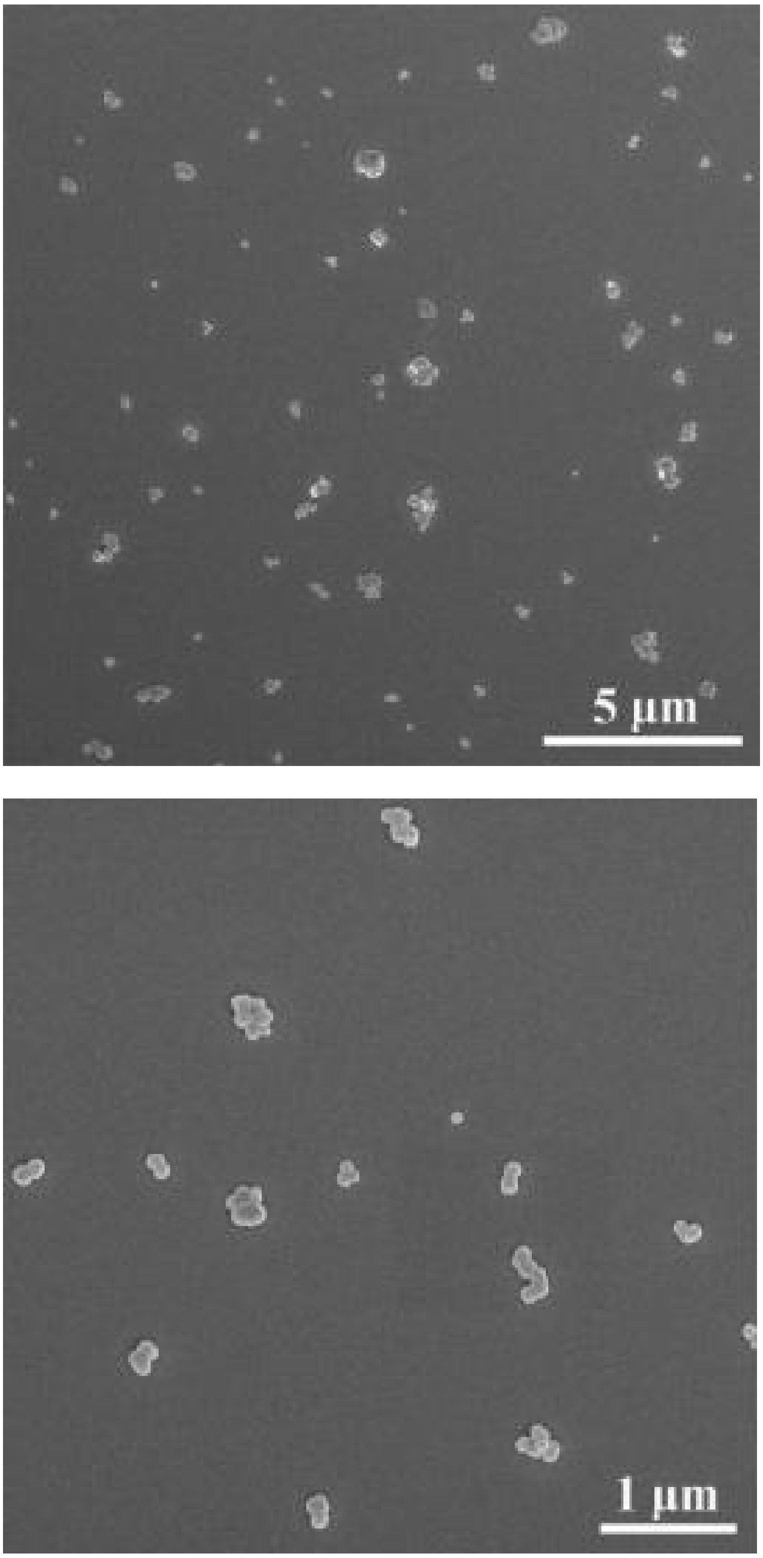

Figure S3. A typical SEM image of phosphoric acid modified BTO.

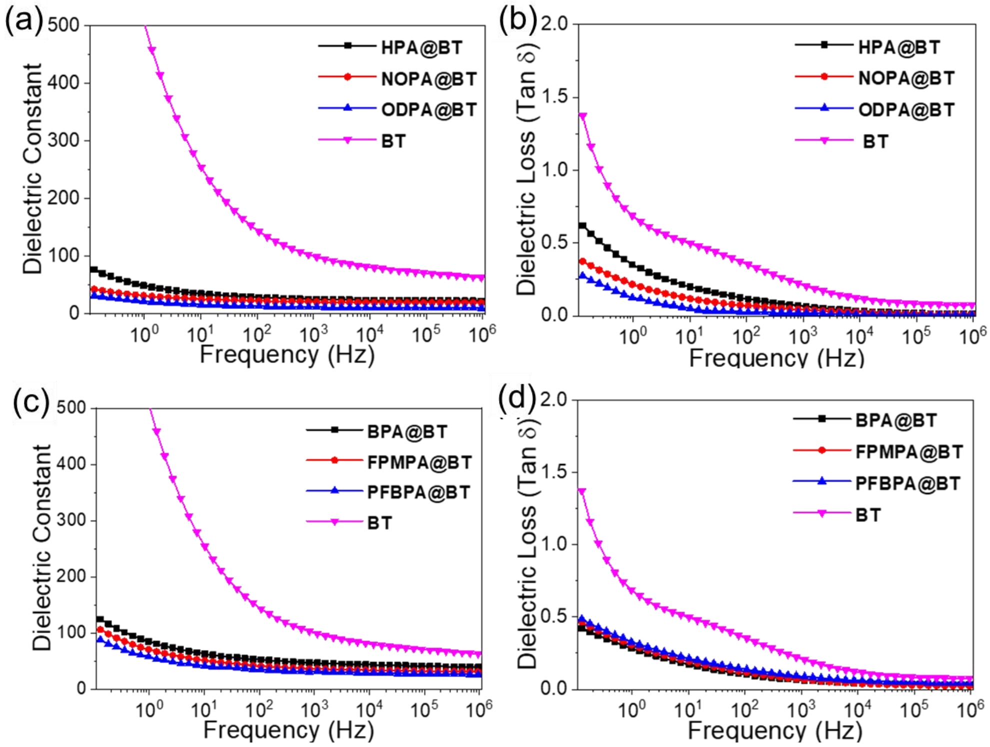

Figure S4. (a) Dielectric constant and (b) dielectric loss of HPA@BTO, NOPA@BTO, ODPA@BTO and unmodified BTO. (c) Dielectric constant and (d) dielectric loss of BPA@BTO, FPMPA@BTO, PFBPA@BTO and unmodified BTO. The frequency range is from 10^{-1} Hz to 10^{6} MHz and all the measurements were performed at room temperature.

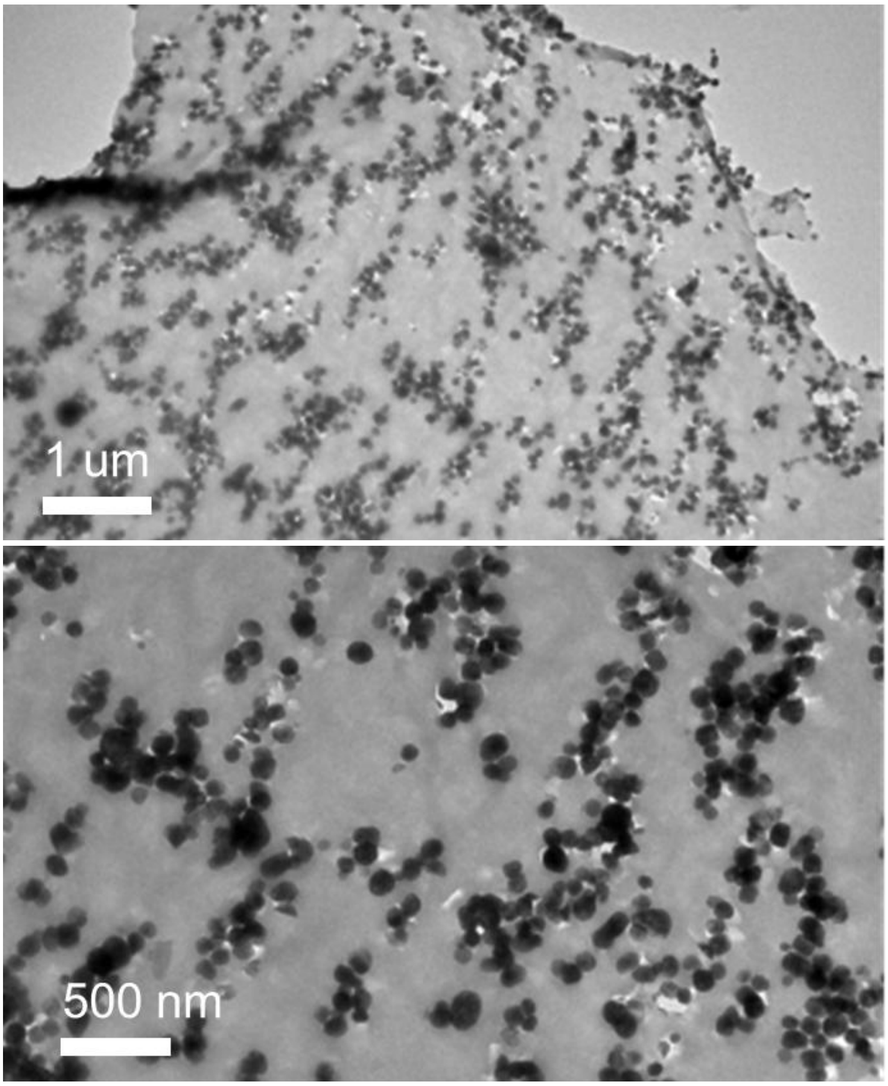

Figure S5. TEM images of ultrathin sections of 20 vol% loading nanocoposites

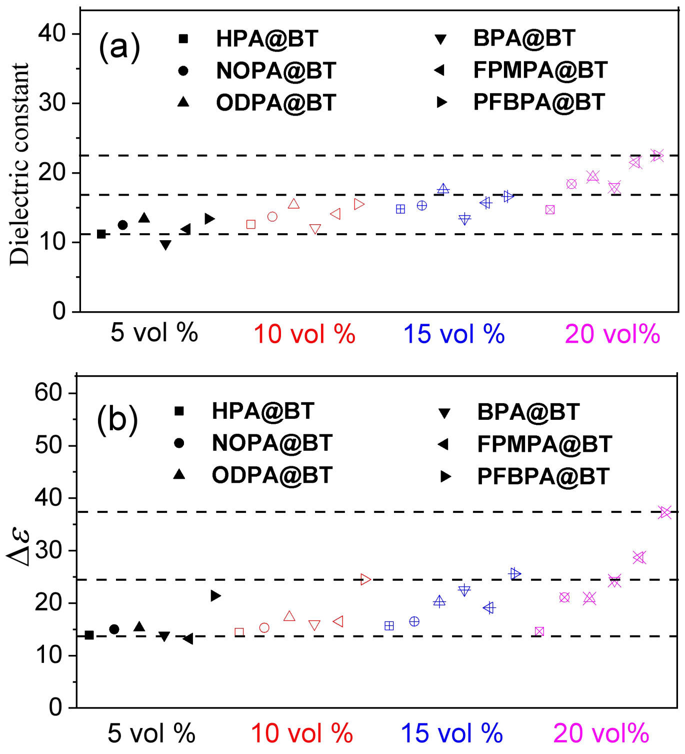

Figure S6. (a) Dielectric constant (ε) and (b) $\Delta\varepsilon$ of nanocomposite films with different fractions of BTO.

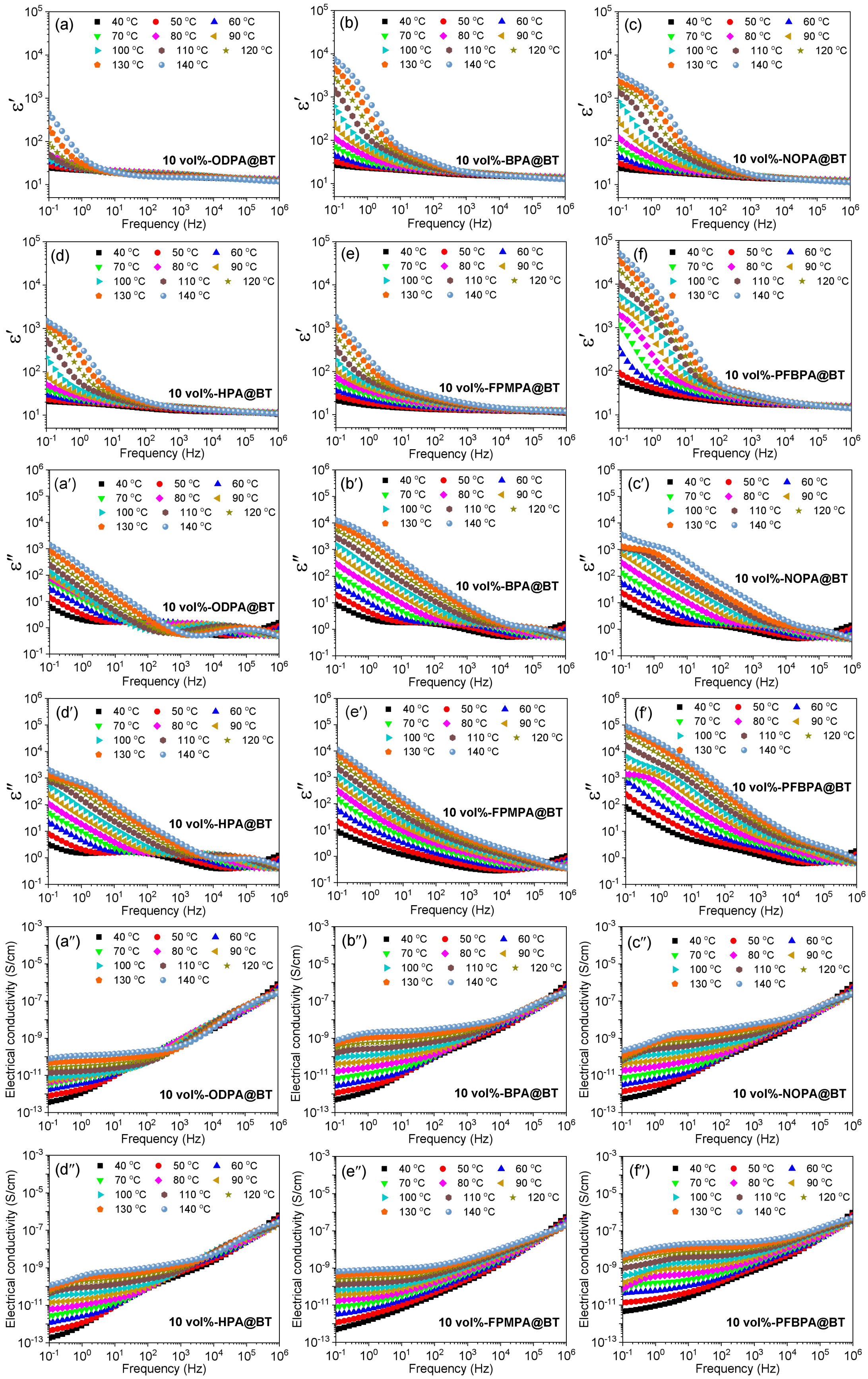

Figure S7. Frequency dependence real dielectric constant (a-f), imaginary dielectric constant (a'-f'), and electrical conductivity (a''-f'') of the P(VDF-HFP)-based nanocomposites with 10 vol% of modified BT nanoparticles.

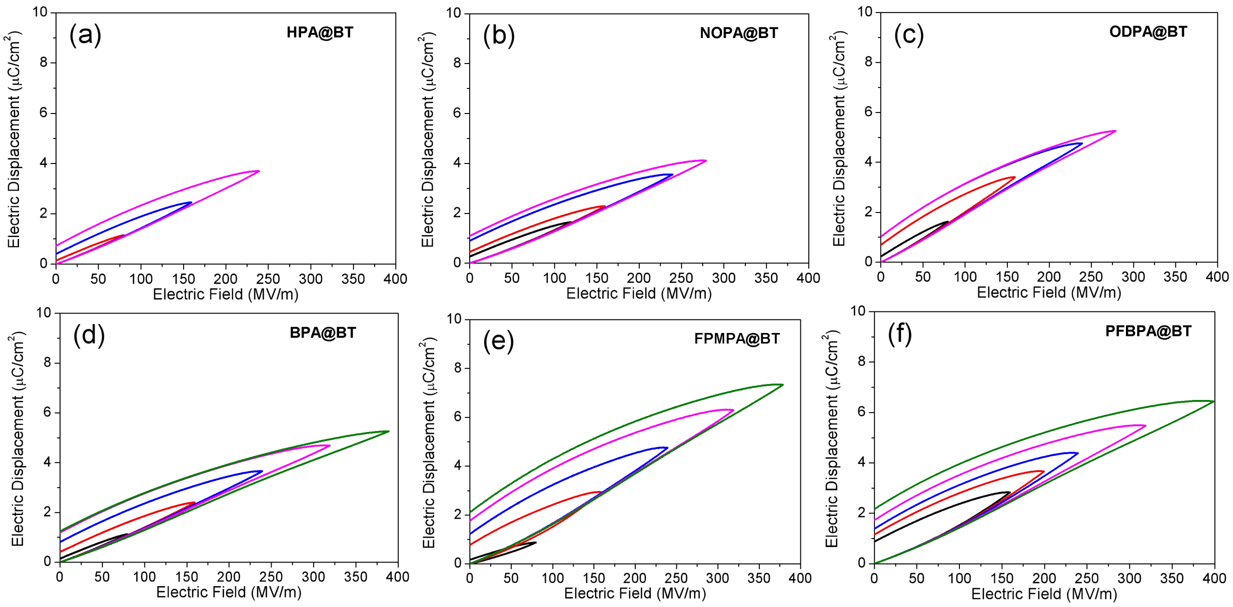

Figure S8. *D-E* loops of the P(VDF-HFP) nanocomposites with 5 vol % modified BT at 100 Hz at room temperature: (a) **HPA@BT/P(VDF-HFP)**, (b) **NOPA@BT/P(VDF-HFP)**, (c) **ODPA@BT/P(VDF-HFP)**, (d) **BPA@BT/P(VDF-HFP)**, (e) **FPMPA@BT/P(VDF-HFP)** and (f) **PFBPA@BT/P(VDF-HFP)**.

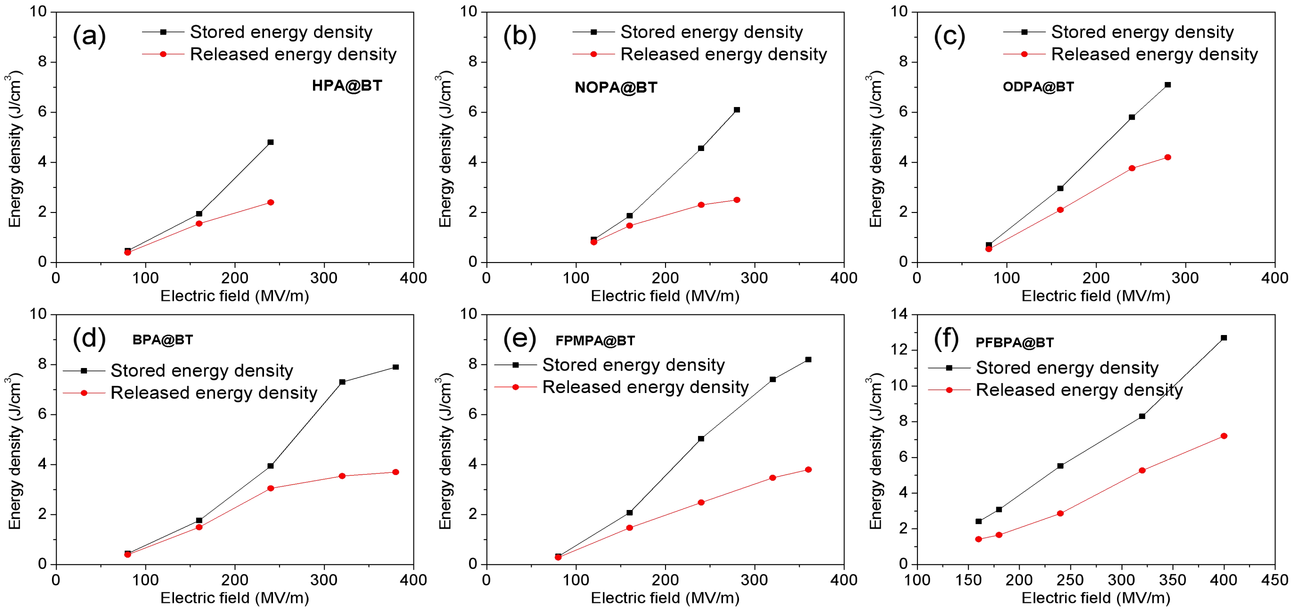

Figure S9. Energy density curves of the P(VDF-HFP) nanocomposites with 5 vol % modified BT at 100 Hz at room temperature: (a) **HPA@BT/P(VDF-HFP)**, (b) **NOPA@BT/P(VDF-HFP)**, (c) **ODPA@BT/P(VDF-HFP)**, (d) **BPA@BT/P(VDF-HFP)**, (e) **FPMPA@BT/P(VDF-HFP)** and (f) **PFBPA@BT/P(VDF-HFP)**.

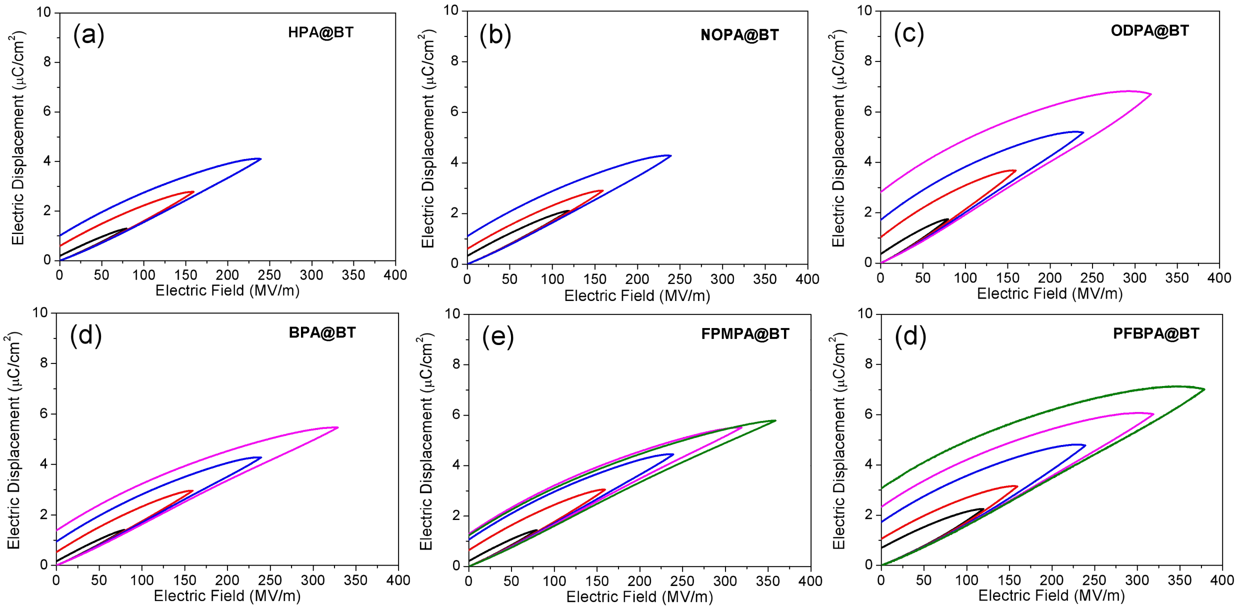

Figure S10. *D-E* loops of the P(VDF-HFP) nanocomposites with 10 vol % modified BT at 100 Hz at room temperature: (a) **HPA@BT/P(VDF-HFP)**, (b) **NOPA@BT/P(VDF-HFP)**, (c) **ODPA@BT/P(VDF-HFP)**, (d) **BPA@BT/P(VDF-HFP)**, (e) **FPMPA@BT/P(VDF-HFP)** and (f) **PFBPA@BT/P(VDF-HFP)**

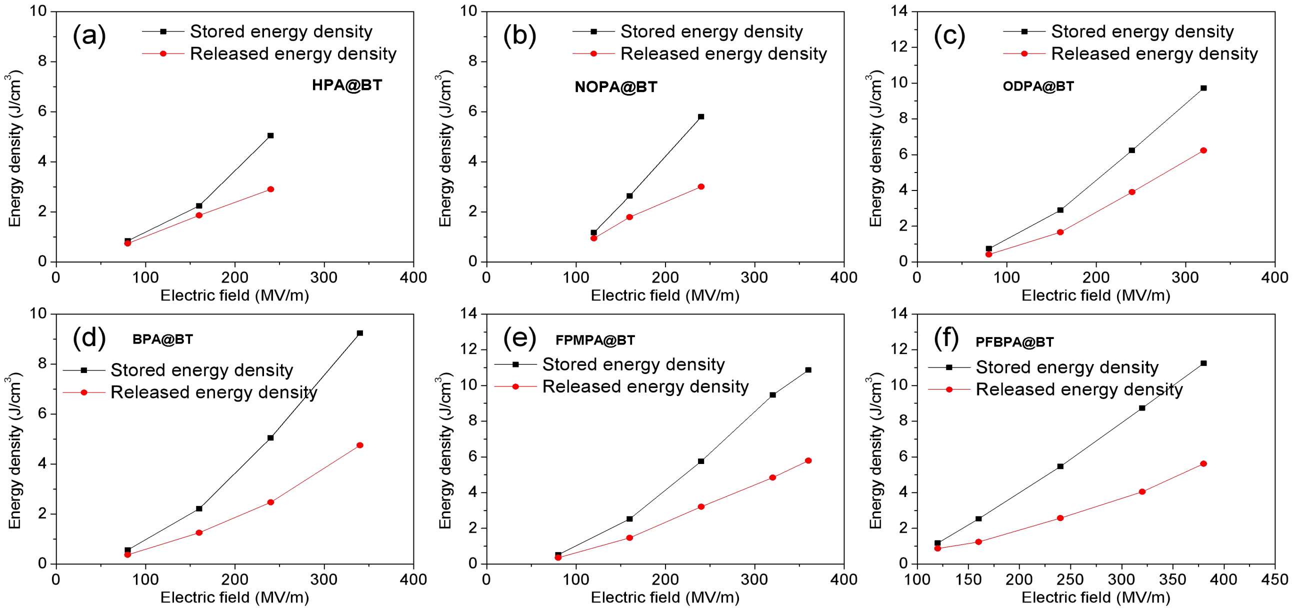

Figure S11. Energy density curves of the P(VDF-HFP) nanocomposites with 10 vol % modified BT at 100 Hz at room temperature: (a) **HPA@BT/P(VDF-HFP)**, (b) **NOPA@BT/P(VDF-HFP)**, (c) **ODPA@BT/P(VDF-HFP)**, (d) **BPA@BT/P(VDF-HFP)**, (e) **FPMPA@BT/P(VDF-HFP)** and (f) **PFBPA@BT/P(VDF-HFP)**.

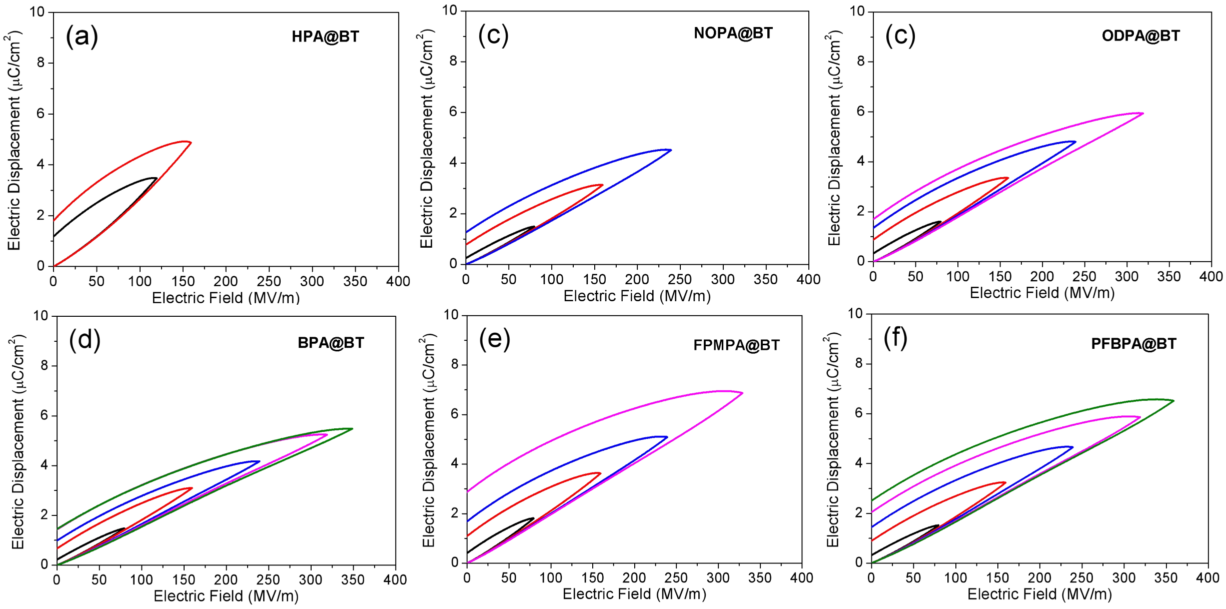

Figure S12. *D-E* loops of the P(VDF-HFP) nanocomposites with 15 vol % modified BT at 100 Hz at room temperature: (a) **HPA@BT/P(VDF-HFP)**, (b) **NOPA@BT/P(VDF-HFP)**, (c) **ODPA@BT/P(VDF-HFP)**, (d) **BPA@BT/P(VDF-HFP)**, (e) **FPMPA@BT/P(VDF-HFP)** and (f) **PFBPA@BT/P(VDF-HFP)**

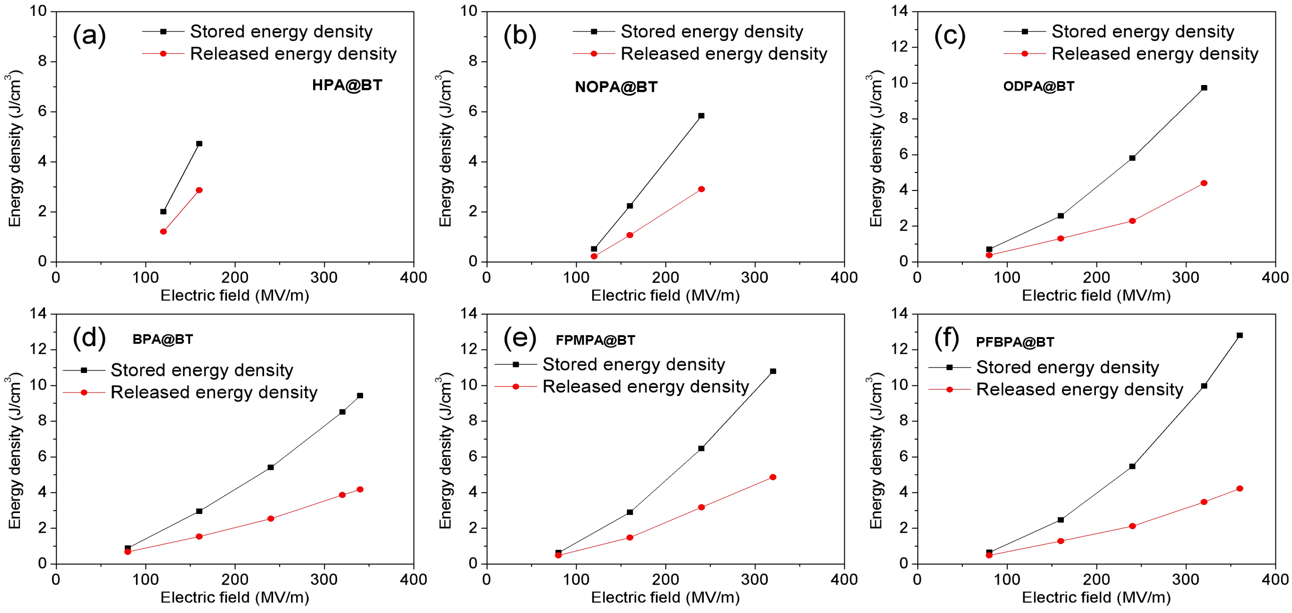

Figure S13. Energy density curves of the P(VDF-HFP) nanocomposites with 15 vol % modified BT at 100 Hz at room temperature: (a) **HPA@BT/P(VDF-HFP)**, (b) **NOPA@BT/P(VDF-HFP)**, (c) **ODPA@BT/P(VDF-HFP)**, (d) **BPA@BT/P(VDF-HFP)**, (e) **FPMPA@BT/P(VDF-HFP)** and (f) **PFBPA@BT/P(VDF-HFP)**.

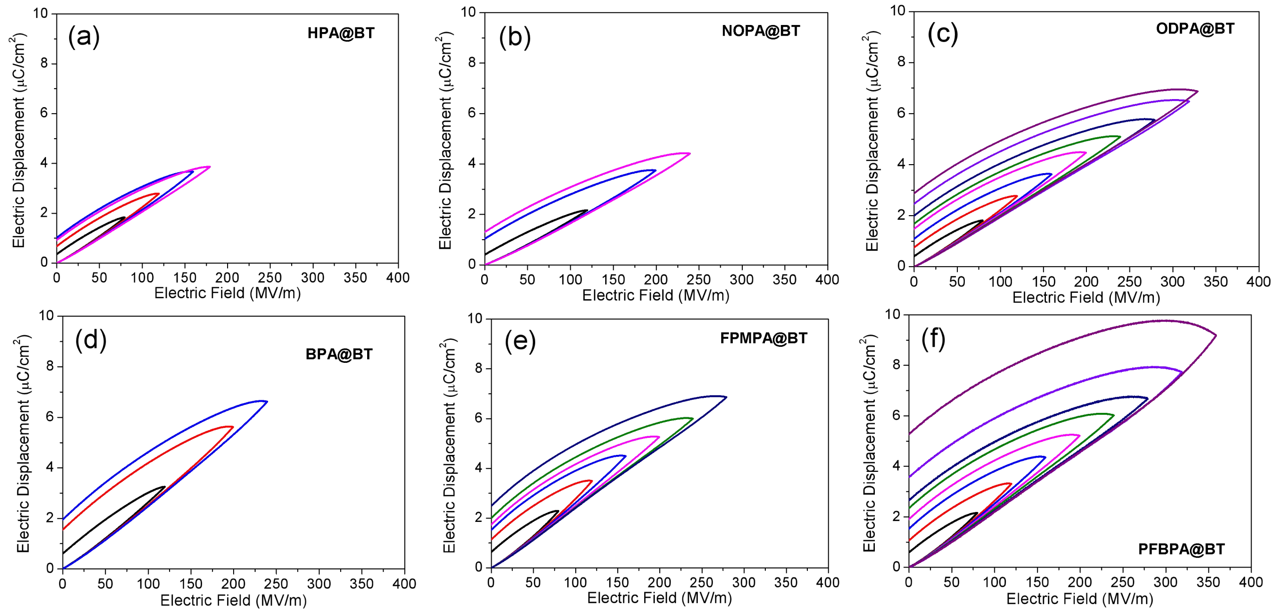

Figure S14. *D-E* loops of the P(VDF-HFP) nanocomposites with 20 vol % modified BT at 100 Hz at room temperature: (a) **HPA@BT/P(VDF-HFP)**, (b) **NOPA@BT/P(VDF-HFP)**, (c) **ODPA@BT/P(VDF-HFP)**, (d) **BPA@BT/P(VDF-HFP)**, (e) **FPMPA@BT/P(VDF-HFP)** and (f) **PFBPA@BT/P(VDF-HFP)**.

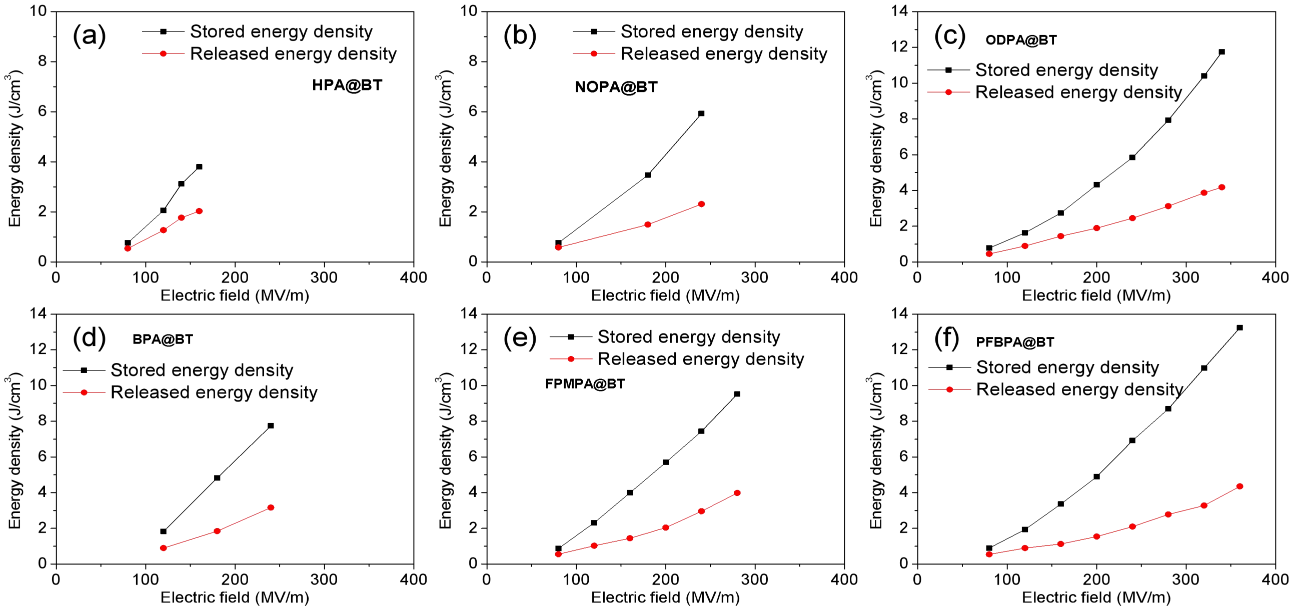

Figure S15. Energy density curves of the P(VDF-HFP) nanocomposites with20 vol % modified BT at 100 Hz at room temperature: (a) **HPA@BT/P(VDF-HFP)**, (b) **NOPA@BT/P(VDF-HFP)**, (c) **ODPA@BT/P(VDF-HFP)**, (d) **BPA@BT/P(VDF-HFP)**, (e) **FPMPA@BT/P(VDF-HFP)** and (f) **PFBPA@BT/P(VDF-HFP)**.
